# A survey of how patient-perceived empathy affects the relationship between health literacy and the understanding of information by orthopedic patients?

**DOI:** 10.1186/1471-2458-13-155

**Published:** 2013-02-19

**Authors:** Cheng-I Chu, Chia-Chih Alex Tseng

**Affiliations:** 1 Department of Public Health, Tzu Chi University, 701, Sec. 3, Zhongyang Rd, Hualien City, Hualien County, 97004, Taiwan; 2 Department of Anesthesiology, Ditmanson Medical Foundation, Chia-Yi Christian Hospital, Chiayi City, Taiwan

**Keywords:** Health literacy, Patient-perceived empathy, Preoperative information understanding

## Abstract

**Background:**

There is a lack of research examining patient-perceived empathy and its effect on low-literacy patients’ understanding of health information. This study investigated the moderating effect of patient-perceived empathy on the relationship between health literacy and understanding of preoperative information.

**Methods:**

During a 2-month period, a total of 144 patients took a survey that included the Chinese-edition Rapid Estimate of Adult Literacy in Medicine, the Barrett-Lennard Relationship Inventory and the Preoperative Information Understanding Scale. Hierarchical multiple regression analysis provided a test of moderator effects.

**Results:**

All Cronbach’s alphas exceeded 0.6, with REALM at 0.91, BLRI at 0.67, and PIUS at 0.77.The finding that the interaction term was significant suggests perceived empathy is a relevant factor when considering the relationship between health literacy and the understanding of information by patients. The relationship between the health literacy and understanding of information was stronger and positive among patients who perceived greater empathy from their physicians.

**Conclusion:**

Our study demonstrates that a focus on improving physician–patient empathy skills could be beneficial in helping to overcome the negative consequences associated with limited health-literacy capabilities. Healthcare providers who wish to improve the understanding of information by low health-literacy patients should first identify components of their empathic communication mechanisms, and then try to refine these skills to better serve their patients.

## Background

Health literacy is increasingly recognized as a critical factor affecting patient-physician communication and health outcomes [1]. The terminology health care providers use to communicate with patients can present a barrier for patients who cannot sufficiently comprehend health vocabulary. The consequences of low health literacy include difficulties navigating the health care system, receiving fewer preventive services, inaccurate or incomplete histories, missed appointments, failing to follow medication instructions, lack of informed consent, and worse health outcomes [2-7].

Patients with low health literacy often do not understand the information given to them. As a consequence, they often feel short of information, which can lead to vagueness, nervousness and anxiety. The literature recommends that patient-centered approaches generally are associated with better feelings of understanding [8]. Of all the factors involved in effective patient-centered communication, empathy seems to be the element most influential, yet also easily ignored [9,10]. Commonly regarded as an essential attribute for doctors [11], empathy is the capacity to understand another person’s experience from within that person’s frame of experience [12]. Empathy is a process involving facets of cognitive, behavioral, and affective actions on the part of physicians [13]. Appropriate use of empathy as a communication tool has multiple benefits in the patient-physician dynamic, including: 1) encouraging patients to better describe their symptoms and concerns; 2) enhancing the efficiency of collecting and understanding health information leading to a more accurate diagnosis; 3) aiding patients in participating in their treatment and recovery; and 4) honoring and soothing patients in a therapeutically beneficial manner [10,14].

Despite the growing research base documenting the extent and impact of low health literacy, there is a lack of research examining patient-perceived empathy and its effect on the understanding of health information by patients with low-literacy. Our study used a sample of orthopedic patients from a medical center in southern Taiwan to investigate the moderating effect of patient-perceived empathy on the relationship between health literacy and understanding of preoperative information.

## Methods

### Participants

All patients visiting the inpatient orthopedic clinic to receive total hip (THA) or knee (TKA) replacement medical services and meeting the eligibility criteria in one of the medical centers in southern Taiwan during the 2-month period from April 15, 2007, to June 15, 2007 were asked to participate. Eligibility criteria were age > 18 years; comfort in speaking Mandarin or Taiwanese; and not having cognitive limitations, mental and sight barriers, or other major diseases. Prior to their decision to participate, patients were given a brief verbal overview of the aim and methods of the study. Participants provided informed consent before they joined the study, which had been previously approved by the Institutional Review Board of Chang Gung Medical Center.

### Data collection

Data on perceived empathy and patient understanding were collected relative to the patient-physician interaction occurring when patients received the medical center’s standard health education materials. A structured questionnaire which included demographic items, Chinese-edition Rapid Estimate of Adult Literacy in Medicine (REALM), Empathy Understanding subscale of the Barrett-Lennard Relationship Inventory (BLRI), and Preoperative Information Understanding Scale (PIUS) was designed by a group of faculty and medical practitioners. Forward and backward translation skills were employed when appropriate to ensure no misinterpretation existed owing to language issues. This is because the original scales came from Western literature. Content validity was assured by means of a careful expert review and a pilot test. Content validity index (CVI) for overall questionnaire from six experts reached 0.85, representing evidence of content validity. Internal consistency reliability coefficients were calculated for the questionnaire. With the help of a well-trained researcher with public health background, health literacy, perceived empathy, preoperative understanding of information, and selected demographic characteristics of the participants were obtained from face-to-face surveys conducted after the patients’ surgery and once the patient was able and willing to answer questions personally.

Health literacy was measured using a Chinese-edition Rapid Estimate of Adult Literacy in Medicine (REALM) test [15]. The REALM was designed to identify patients who may need help with health care instructions because of low literacy [16]. Fisher asserted that REALM is useful for screening individuals who are at risk because of their inability to read [17]. It measures a patient’s ability to read and correctly pronounce 66 common medical terms that patients might encounter, with the total number of correctly pronounced words constituting each patient’s REALM score. The 66 common medical terms in the Chinese-edition of the REALM were chosen by a group of health care researchers in their study of different levels of health literacy. Each medical term is scored as 0 for “did not pronounce well” and 1 for “pronounced well”; such that the maximum score for health literacy is 66. The Cronbach’s alpha value of the Chinese-edition REALM was lacking in literature.

Perceived empathy was measured using the 16-item Empathy Understanding subscale of the Barrett-Lennard Relationship Inventory (BLRI), which has been robustly used in psychotherapy outcome research and had several advantages over other available instruments: patient centered, appropriate validity and reliability, uses multiple items, inexpensive and free of technical complexity [18]. All items in the perceived empathy assessment were scored on a five-point Likert scale, with 1 = strongly disagree, 2 = disagree, 3 = neutral, 4 = agree, and 5 = strongly agree, with a total score of 80 for BLRI. Higher scores represented more empathy perceived by patients. Gurman (1977) found that BLRI had good internal consistency with alpha of 0.84 for empathy.

Understanding of preoperative information was measured using an 18-item researcher-developed Preoperative Information Understanding Scale (PIUS) based on suggestions from a preoperative teaching questionnaire and the literature [19-21]. All participants were assessed for understanding of preoperative information in three areas: operation information (5 items), anesthesia information (4 items), and nursing instructions information (9 items). A review by experts ensured content validity, and reliability of the measures was assured for each area by having Cronbach’s alphas ≥ 0.7. Responses for each area were scored numerically and divided into four levels of understanding: information not provided (0), information provided but not understood (1), information provided and partly understood (2), and information provided and well understood (3). A higher total score represented better understanding of preoperative information.

### Statistical analyses

Statistical analyses were conducted with the Statistical Package for the Social Sciences (SPSS). We used a number of statistical methods to detect moderator variables. The general strategy is to test for an interaction using hierarchical multiple regression analysis [22]. In the first step, we entered the independent variables (including the control variables and the moderator) into the regression model to verify the main effects of the independent variables. Then, in a separate step, the product of the independent variables, which represents the moderator effect, was entered. Note that in order to eliminate the effect of multicollinearity of variables, the product item was formed by multiplying together the two centered variables (i.e., put in deviation score form so that their means are zero): perceived empathy and health literacy [23,24]. Cohen had stated that this hierarchical approach provides an unambiguous test of moderator effects [25]. To further describe the moderating effect, the subgrouping strategy of Arnold was executed to show the strength or degree of relationship between a moderator variable (i.e., perceived understanding) and another variable (i.e. health literacy) in predicting values of a third variable (i.e., understanding of information) [26]. We used the median of perceived empathy, which was 52 out of 80, to divide participants into two subgroups [24]: one for a “high” perceived empathy score (≥52) and one for a “low” perceived empathy score (<52). The slopes of the regression lines from these subgroups were plotted to identify the form of the moderator effect [27].

## Results

Of the 191 eligible patients, a total of 144 agreed to participate and finish the survey during a 2-month period. The results indicated that all Cronbach’s alphas exceeded 0.6, with REALM at 0.91, BLRI at 0.67, and PIUS at 0.77. Table 1 shows demographic data for the participants. Participants were generally older (72.2% aged >50 years), with an average age of 57.3 years in a range of 20–87 years. Of the participants, 86 (59.7%) were women and 58 (40.3%) were men. Nearly 60% were illiterate or had only primary school education. Most were married (104, 72.2%) and the majority stated their religious affiliation as Buddhist (61, 42.4%) or Taoist (60, 41.7%). 

**Table 1:** Demographic characteristics of participants (N = 144)

**Variable category**	**n (%)**
Age,y	
≦50	40 (27.8%)
>50	104 (72.2)
Sex	
Male	58 (40.3)
Female	86 (59.7)
Education level	
Illiteracy	35 (24.3)
Primary school	50 (34.7)
Junior high	12 (8.3)
Senior high	27 (18.8)
College/University	20 (13.9)
Marital status	
Single	20 (13.9)
Married	104 (72.2)
Widowed	20 (13.9)
Religion	
None	16 (11.1)
Buddhist	61 (42.4)
Taoist	60 (41.7)
Others	7 (4.9)

We performed a two-step hierarchical multiple regression analysis to more fully understand the moderating effect of perceived empathy. Table 2 demonstrates the moderating effect of perceived empathy on the association between health literacy and understanding of information, controlling for the demographic variables. We entered demographic variables (age as a continuous variable; sex, marital status, education level, and religion as dummy variables), perceived empathy, and health literacy as the predictor variables in Step 1. Then, we added the two-way interaction between centered perceived empathy and centered health literacy as the predictor variable in Step 2. The finding that the interaction term was significant suggests the moderator (i.e., perceived empathy) is a relevant factor when considering the relationship between health literacy and the understanding of information by patients. 

**Table 2:** Results of hierarchical regression analyses

	**Predictor variables**	**Information understanding**		
		**b**^**1**^	**β**^**2**^	**t**^**3**^
Step1:				
	Age	-.108	-.196	−1.737
	Female	.433	.024	.275
	Married	1.299	.066	.749
	Primary school	.562	.031	.243
	Junior high	−2.049	-.065	-.573
	Senior high	2.397	.107	.667
	College University	4.214	.166	1.088
	Buddhist	-.884	-.050	-.337
	Taoist	−3.412	-.192	-.1296
	Others	−3.039	-.075	-.760
	Perceived empathy	-.356	-.143	−1.640
	Health literacy	.020	.066	.500
Step 2:				
	Age	-.116	-.211	−1.910
	Female	.193	.011	.126
	Married	-.837	.043	.492
	Primary school	.702	.038	.311
	Junior high	−2.116	-.067	-.606
	Senior high	2.360	.105	.673
	College University	4.508	.178	1.192
	Buddhist	−1.936	-.109	-.748
	Taoist	−4.277	-.240	−1.652
	Others	−5.039	-.124	−1.270
	Perceived empathy	-.488	-.196	−2.245*
	Health literacy	.012	.040	.307
	Perceived empathy × Health literacy	.019	.225	2.740**

^1^ unstandardized coefficient ^2^ standardized coefficient ^3^t values * p<.05 ** p<.01.

**Figure 1:**
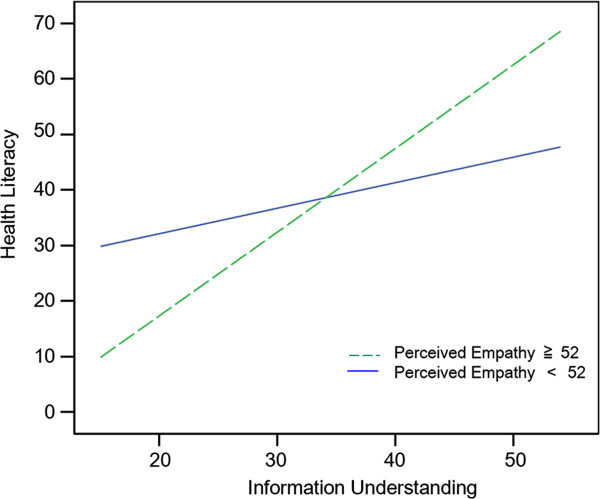
Graphical presentation of the interaction between perceived empathy and health literacy in predicting information understanding.

**Figure 2:**
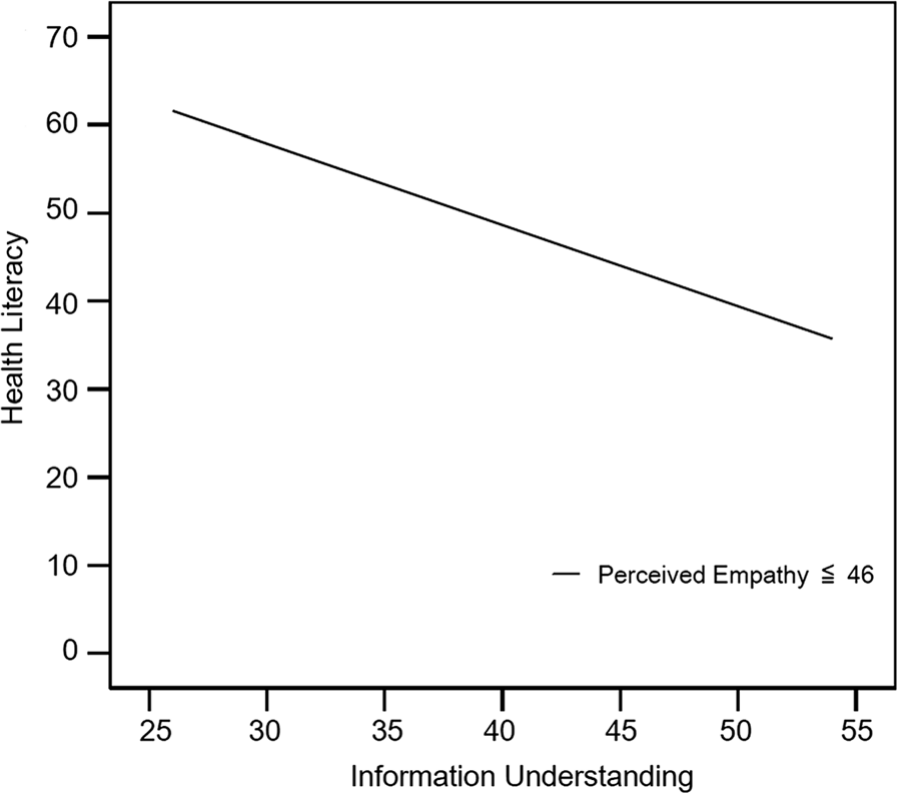
Graphical presentation of the slope change between the health literacy and information understanding among patients who perceive low empathy.

In order to identify the form of the moderator effect, two slopes were graphically plotted [27], one for a “high” perceived empathy score (≥52, the median) and one for a “low” perceived empathy score (<52). As shown in Figure 1, the graph indicates that the relationship between information understanding and health literacy was stronger and positive among patients who perceived higher empathy from their physicians. The relationship was weak and positive among patients who perceived low empathy from their physicians. However, in Figure 2, the relationship became negative among the lowest 10% perceived empathy score patients (≤46, N = 14). 

## Discussion

The results of the two-step hierarchical multiple regression analysis indicated that patients’ perceived empathy from their physicians interacted with the relationship between health literacy and their understanding of information. The graph of the regression lines verified the moderator effect of perceived empathy. For patients whose perceived empathy was greater, their understanding of information exhibited stronger positive relationships with their health literacy. In other words, more empathy from physicians may help patients understand more preoperative information regardless of patients’ low level of health literacy. Patients who perceived comparatively low empathy from their physicians exhibited an opposite pattern of relationship between health literacy and understanding of information. Low empathy from physicians may negatively affect patients’ understanding of preoperative information without regard to their level of health literacy. Research has revealed that when opportunities for empathy are repeatedly ignored or missed, visits tend to be more time-consuming and frustrating for both physicians and patients [28,29]. The findings of the present study further demonstrate that, in addition to facilitating the clinical interview and honoring the patient, empathy may improve the efficiency of understanding information.

Though researchers have cautioned not to overemphasize reading level scores by overlooking other factors that can affect the ability to read, such as motivation, interest, need, culture, acute versus chronic illness, physical limitations, and cognitive limitations [30], we found many patients’ low health-literacy levels resulted from never having attended school, or having only attended primary school. As it is not possible to improve literacy levels in a short time, it is worth noting that improving empathic communication skills among healthcare providers can provide a feasible means to increase patients’ understanding of health information during a visit. Owing to poor health literacy often being associated with a lack of medical knowledge, inferior health status, and a higher use of medical services, efforts ought to be undertaken to address the needs of populations with limited health literacy. With the intention of altering the old-world view that physicians are trained in a system where empathetic communication is only an afterthought, many medical schools have developed curricula with a solid focus on physician–patient communication and empathy.

Our study has several limitations. First, the data may be subject to common method variance as they all came from a unique source. Second, replication in other medical departments is desirable to allow a more accurate appraisal. Third, other factors were not measured and controlled but may have affected the relationship between health literacy and understanding of information (e.g., patient participation). Fourth, studies are needed to replicate these findings using alternative measures of health literacy and perceived empathy because different measures may yield different results. Finally, further evaluation on the validity and reliability of the translated questionnaire is highly recommended so as to assure the tools used are reliable and have adequate validity.

## Conclusions

A variety of methods have been advocated for communicating with patients who have low literacy skills. Whereas there is abundant research documenting the prevalence of low health literacy and its correlation with insufficient health knowledge, and less-optimal health outcomes, our study shows that a focus on improving physician–patient empathy skills could be substantially beneficial in helping to overcome the negative consequences associated with limited health-literacy capabilities. Dealing with a patient who has low literacy takes time and money. Our study demonstrates that patients with higher perceived empathy exhibited stronger understanding of preoperative information.

Healthcare providers who wish to improve the understanding of information by low health-literacy patients should first identify components of their empathic communication mechanisms, and then try to refine these skills to better serve their patients.

## Competing interests

We certify that the submitted manuscript poses no financial and non-financial competing interests over the past five years and for the foreseeable future.

## Authors’ contributions

CIC was the lead writer and coordinator on the manuscript, and worked on the content development and distribution of the survey instrument. CCT contributed to writing survey instrument content, coauthored the Background, Discussion, and Conclusions sections, and co-edited the manuscript. All authors read and approved the final manuscript.

## Pre-publication history

The pre-publication history for this paper can be accessed here:

http://www.biomedcentral.com/1471-2458/13/155/prepub
